# Traditional Chinese medicine-related interventions and the cGAS–STING axis in neuroinflammation: disease contexts and mechanisms of action

**DOI:** 10.3389/fneur.2026.1979957

**Published:** 2026-10-16

**Authors:** Cunlong Zhang, Qingqing Li

**Affiliations:** Pengzhou Hospital of Traditional Chinese Medicine (Peony New City Campus), Pengzhou, China

**Keywords:** acupuncture, cGAS-STING, microglia, mitochondrial DNA, neuroinflammation, traditional Chinese medicine, type I interferon

## Abstract

Research on traditional Chinese medicine (TCM)-related interventions has shifted from overall phenotypic effects to defined neuroimmune targets. The cyclic GMP-AMP synthase-stimulator of interferon genes (cGAS-STING) axis connects cytosolic DNA sensing with host defense and sterile inflammatory injury, but the functional direction of this pathway changes with disease context. This review organizes preclinical findings by pathological setting and site of action. It examines herbal formulas and natural compounds, followed by acupuncture, electroacupuncture, and related delivery systems. Several sterile injury models associate benefit with reduced pathway-related inflammation. Acute sleep deprivation provides an example of protection accompanying restored STING signaling. Early herpes simplex virus infection follows a different pattern: restoration of suppressed antiviral signaling improves host defense. In acute postoperative pain and cancer-induced bone pain models, increased STING-IFN-I signaling accompanies electroacupuncture-induced analgesia. Some interventions reduce mitochondrial DNA stimulation. Others act on cGAS or STING, with separate studies targeting STING trafficking. Downstream inflammatory and cell-death responses form the final level. Mechanistic attribution is strongest when genetic or pharmacological perturbation links pathway change to functional outcome; direct binding has been demonstrated for a small group of compounds. These findings support a context-sensitive framework for interpreting modulation direction that requires validation within individual disease models. Clinical translation will depend on reproducible formulation composition, effective brain exposure, and safety.

## 1 Introduction

Neurological disorders impose a growing burden of disability (1). Neuroinflammation is common across these disorders and can be both protective and injurious. Innate immune responses support host defense and tissue adaptation, whereas persistent activation aggravates neuronal and vascular injury (2, 3). This duality complicates broad anti-inflammatory treatment and shifts attention toward the initiating signal, the responding cell, and the stage of disease.

The cGAS-STING axis links cytosolic DNA sensing to neuroinflammation. cGAS recognizes double-stranded DNA and synthesizes 2′3′-cGAMP, which activates STING. STING then engages TBK1-IRF3 signaling, while NF-κB drives a parallel inflammatory response. These pathways induce type I interferons and inflammatory cytokines (4). During sterile neural injury, mitochondrial damage releases mtDNA into the cytosol. Micronuclear rupture provides another source of self-DNA, while defective clearance prolongs its cytosolic presence (5–8). The pathway thus translates cellular and metabolic stress into innate immune activation.

The consequences of cGAS-STING activation depend on disease context. Constitutive STING activity induces neuroinflammation and dopaminergic neurodegeneration in mice (9). Genetic or pharmacological inhibition is protective in selected models of aging and proteinopathy (10–13). By contrast, effective STING-type I interferon signaling must be preserved during early antimicrobial defense, and functional outcomes in pain studies vary with the neuronal or glial compartment involved (14–17). These differences indicate that the direction of cGAS-STING modulation should vary across disease contexts.

Reviews of neurodegeneration discuss cGAS-STING among several neuroimmune pathways (18). TCM-related studies have examined this axis in specific experimental settings, including tetramethylpyrazine treatment in cerebral ischemia models (19). This review organizes the literature along two independent dimensions. Pathological context provides a basis for comparing restriction, preservation, and restoration of pathway activity. Site of action identifies where an intervention enters the pathway, from cytosolic DNA input to cGAS or STING regulation and subsequent inflammatory output. We separately assess how each mechanistic claim was established, distinguishing pathway-associated changes from perturbation experiments and direct molecular interactions. This framework allows herbal medicines, natural compounds, and acupuncture-based interventions to be compared without assuming a uniform target or direction of modulation.

## 2 Literature search and study selection

Relevant literature was identified through PubMed searches conducted up to the time of manuscript preparation, without a formal lower date limit or predefined language restriction. Search terms combined concepts related to cGAS-STING, neuroinflammation, neurological disorders, Chinese herbal medicine, natural compounds, acupuncture, and electroacupuncture. Additional searches used the names of compounds, herbal formulas, and pathway modulators identified during screening. Reference lists of key reviews and relevant primary studies were also screened. Because this was a narrative review, study selection was purposive and non-exhaustive, with emphasis on representative findings across disease contexts and intervention types.

## 3 cGAS-STING signaling in neuroinflammation

### 3.1 Signaling architecture and regulatory nodes

The cyclic GMP-AMP synthase-stimulator of interferon genes (cGAS-STING) pathway converts cytosolic DNA sensing into type I interferon (IFN-I), inflammatory, and antiviral responses (20–22). cGAS binds double-stranded DNA without sequence specificity (23, 24). DNA binding opens the catalytic domain of cGAS, enabling ATP and GTP to be converted into the cyclic dinucleotide 2′3′-cGAMP, the endogenous second messenger of the pathway (4, 25). DNA engagement also promotes liquid–liquid phase-separated cGAS condensates that concentrate the enzyme and substrate, whereas divalent manganese lowers the catalytic threshold. Sensor activity is therefore regulated by ligand abundance, the physical state of cGAS, and the local ionic environment (26).

cGAMP activates the endoplasmic reticulum adaptor STING (27). STING then traffics through the ER-Golgi intermediate compartment and recruits TANK-binding kinase 1 (TBK1), which sequentially phosphorylates STING and IRF3 (28). Phosphorylated IRF3 dimerizes and enters the nucleus, where it cooperates with NF-κB to drive transcription of IFN-I, interferon-stimulated genes, and proinflammatory cytokines (29, 30). The interferon-stimulated response also includes protein ISGylation programs implicated in central nervous system disorders (31). STING trafficking controls both signal initiation and termination: the membrane route that permits TBK1-IRF3 signaling also directs STING toward lysosomal degradation within the connected mitochondrial, ER, Golgi, and lysosomal network (32). Potential intervention points therefore extend from the sensor, second messenger, and adaptor trafficking to downstream kinases and transcriptional outputs (Figure 1).

**Figure 1:**
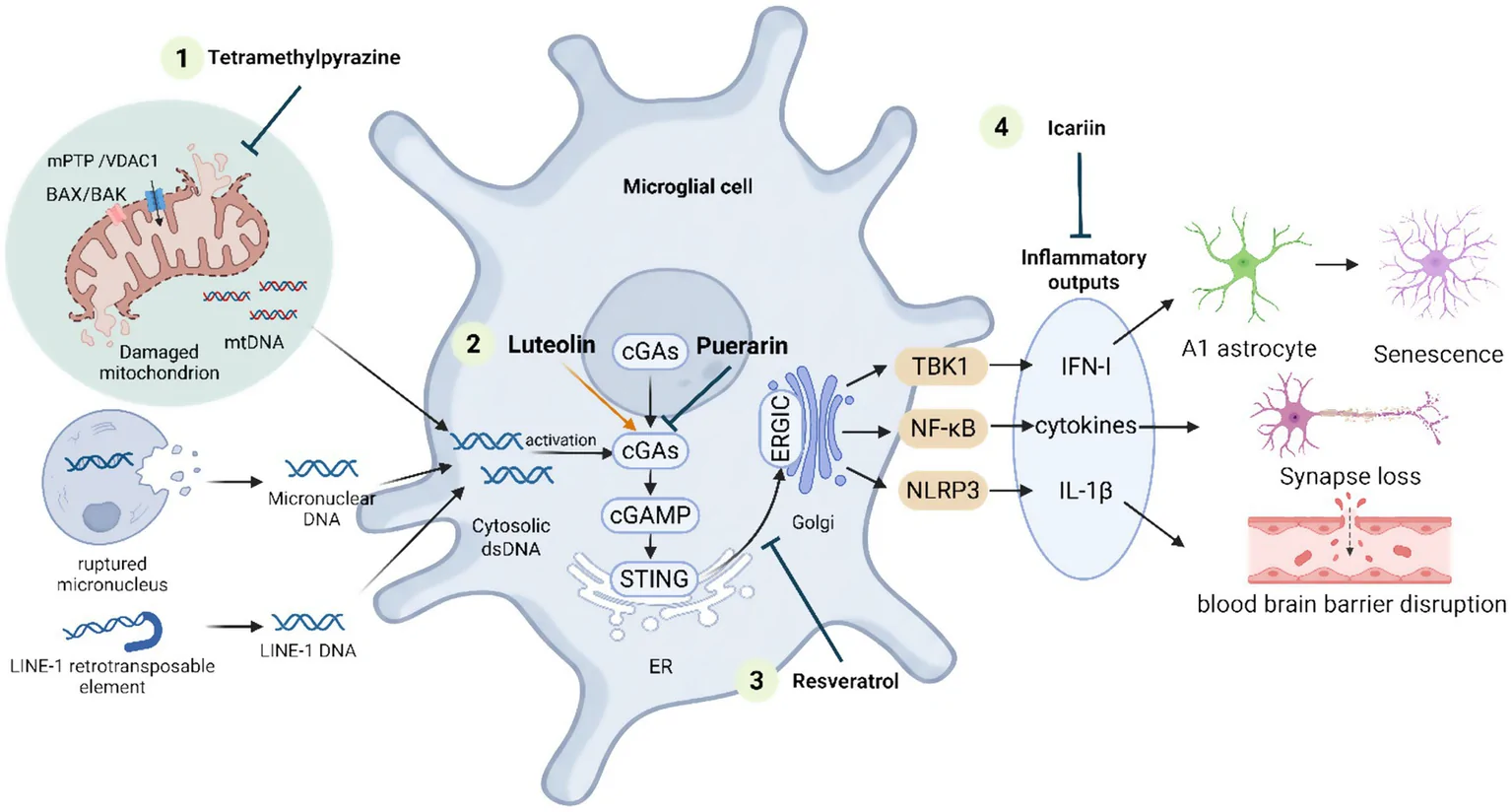
Reported multi-node modulation of the microglial cGAS-STING axis by TCM-related interventions. Cytosolic mtDNA, micronuclear DNA, and LINE-1-derived DNA activate cGAS-cGAMP-STING signaling and connect to TBK1-IFN-I, NF-κB-cytokine, and NLRP3-IL-1β outputs. Numbered sites denote levels of action reported in preclinical studies: ① tetramethylpyrazine reduces mtDNA release; ② the proposed puerarin-cGAS relationship is based mainly on molecular docking, whereas luteolin has biochemical binding results; ③ resveratrol affects ER-Golgi trafficking; and ④ icariin reduces inflammatory output. These positions indicate the reported level of action, and the degree of direct target support for each intervention should be interpreted according to the corresponding study design. Black arrows denote pathway relationships, teal blunt-ended lines denote inhibition, and orange arrows denote activation.

Two additional considerations are relevant in brain tissue. First, STING signaling includes functional branches beyond IFN-I. STING-bearing membranes can initiate autophagic and lysosomal gene programs, linking the pathway to both inflammation and proteostasis (33–35). This ancient autophagic branch is considered protective because clearance of damaged organelles and aggregated proteins removes stimuli that would otherwise sustain signaling (32). Second, nuclear cGAS is restrained by histone tethering under homeostatic conditions (21). Nuclear cGAS can also suppress homologous recombination in non-neuronal experimental systems (36). DNA-repair defects have been linked to neurodegeneration (37), but the contribution of this nuclear cGAS mechanism to postmitotic neurons requires separate investigation. By comparison, the cytosolic IFN-I/NF-κB branch has a more substantial research basis in the central nervous system.

### 3.2 Endogenous DNA sources and cytosolic release

Endogenous DNA is the main pathway trigger in neurodegenerative models. Mitochondrial stress releases mtDNA into the cytosol and induces an antiviral-like innate immune response (38, 39). This route links bioenergetic decline in the aging brain to sterile inflammation (40, 41). Permeability transition and VDAC oligomerization can open mitochondrial exit routes. BAX/BAK pores provide another route, and mitochondria-ER contact sites modify the release process (7, 42). The relative contribution of each route varies with the injury model. Cytosolic mtDNA activates cGAS and can also engage inflammasomes. TLR9 coactivation can further alter the response (43). Parallel activation means that downstream changes cannot be assigned to cGAS without pathway perturbation.

In models of amyotrophic lateral sclerosis (ALS), TDP-43 proteinopathy shows this sequence. Excess TDP-43 enters mitochondria, opens membrane pores, and releases mtDNA before cGAS-STING activation. Blocking mtDNA escape or the downstream sensor reduces neural injury (11). Defective mitophagy lowers the activation threshold by leaving damaged organelles in place (44–47). This places impaired mitochondrial clearance between bioenergetic decline and persistent DNA sensing in the aging brain. The intensity of mitochondrial stress changes the balance between adaptive clearance and inflammatory injury (48).

Nuclear instability and retroelement activity provide additional innate immune stimuli. Ruptured micronuclei expose chromatin to cGAS (5). Age-related LINE-1 derepression can generate cytosolic LINE-1 cDNA that activates cGAS-STING (21). Endogenous retroviral reactivation involves distinct innate immune mechanisms, including RNA-sensing pathways (49, 50). STING activity can also persist without canonical cGAS input. In C9orf72 loss-of-function models, defective degradation prolongs STING signaling (51, 52). TCM-related interventions consequently act at distinct points. Some reduce DNA release, while others change sensor activation. STING trafficking and pathway clearance form separate control levels.

### 3.3 Cellular context and functional outcomes

Microglia are major responders to cGAS-STING activation in the brain. Persistent activation induces inflammatory states whose interferon and cytokine outputs affect neighboring cells (53, 54). A proposed model of postoperative microglial inflammatory memory links cGAS-STING signaling to epigenetic remodeling and sustained priming (55). Lipid-droplet-accumulating microglia have been described as a metabolic state associated with aging and neurodegeneration; cGAS-STING amplification forms part of the proposed inflammatory network (56). Astrocytes also express cytosolic DNA sensors and contribute to DNA-triggered inflammatory responses (57).

The effects of neuronal signaling are more context dependent. Through type I interferon signaling, STING regulates sensory-neuron excitability and nociception (58), so the preferred direction of modulation in pain must be interpreted according to the predominant responding compartment. At the vascular interface, endothelial cGAS-STING can disrupt the blood–brain barrier and promote leukocyte entry, linking the pathway to barrier injury in stroke and hemorrhage (59). Transfer of mtDNA and cGAMP between compartments may also propagate activation from stressed neurons to glial cells (20).

The clearest pathological roles of brain cGAS-STING come from studies of persistent activation or direct pathway perturbation. Constitutively active STING induces α-synuclein pathology, neuroinflammation, and subsequent neurodegeneration in mice, whereas STING blockade or deletion reduces pathology in selected models of aging and proteinopathy (9, 60). These findings locate a pathogenic role for sustained signaling in several sterile neurodegenerative models, while its preferred direction remains dependent on disease stage and cell type (61). Associations of STING with NLRP3, apoptosis, pyroptosis, and ferroptosis indicate that it can amplify injury-related outputs (62–64). A unified PANoptosis program, bidirectional ferroptosis circuitry, and the quantitative contribution of this pathway to inflammaging remain priorities for direct validation (65, 66).

Protective effects have been reported in defined defense and injury settings. Transient activation supports antiviral responses against neurotropic infection (22), whereas STING-IFN-I signaling in pain circuits can have pro- or antinociceptive effects (17). These observations motivate model-specific assessment of the direction of cGAS-STING modulation. Signal duration and responding cell type are candidate explanations for divergent outcomes that should be tested in subsequent preclinical studies (Figure 2).

**Figure 2:**
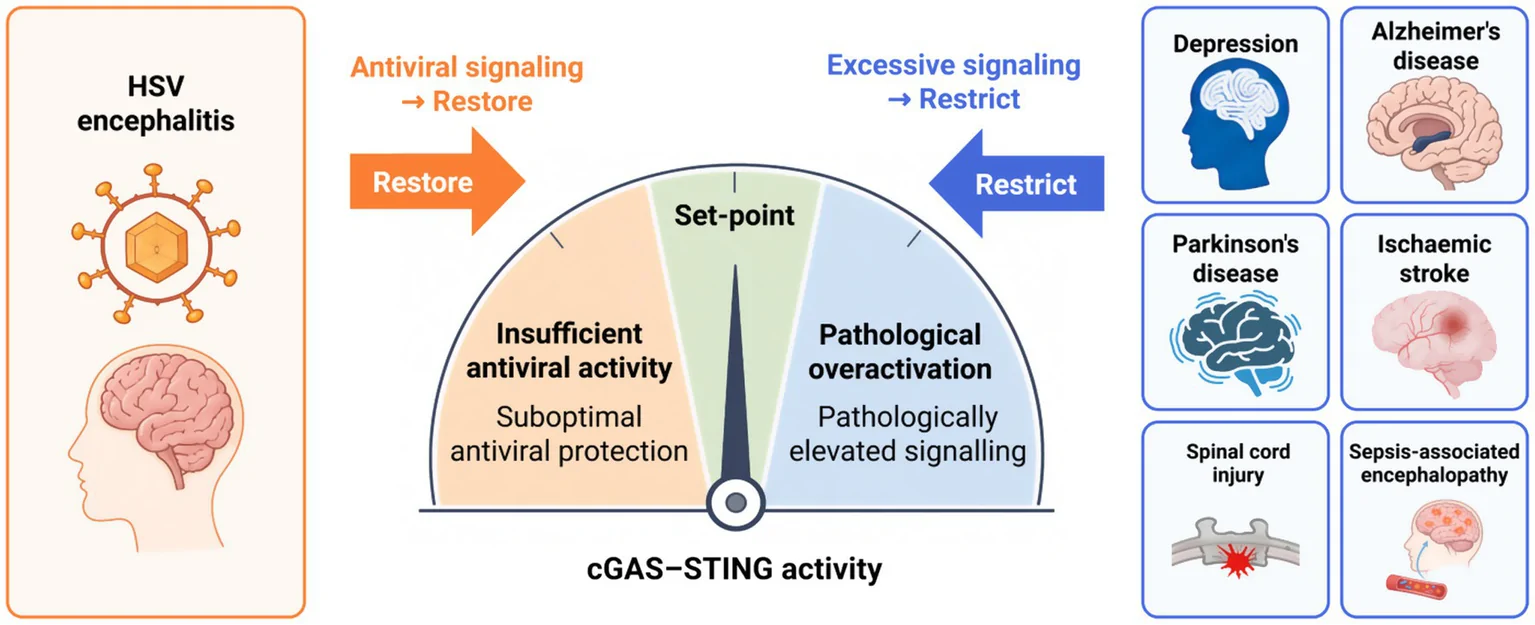
Conceptual model of context-dependent cGAS-STING modulation in central nervous system disorders. The dial represents a conceptual balance rather than a measured physiological or therapeutic threshold. Restoration of suppressed early signaling is protective when antiviral defense is inadequate, whereas restriction of excessive cGAS-STING-related output is more often associated with benefit in models dominated by sterile injury or postinfectious inflammatory damage. These differences are derived from current preclinical observations; subsequent experiments should test the explanatory roles of treatment timing and responding cell type. Orange, blue, and green denote activation, inhibition, and the conceptual set point, respectively.

## 4 Patterns of cGAS-STING modulation across disease contexts

Cross-disease comparison identifies recurring patterns and model-specific differences. Several models of cerebral ischemia, neurodegenerative disorders, postoperative dysfunction, and spinal cord injury associate benefit with reduced cGAS-STING-related injury output. Acute sleep deprivation instead shows protection accompanying restored signaling (67). Early viral infection more often favors restoration of suppressed antiviral signaling. The acute postoperative pain and cancer-induced bone pain studies also report activation-associated analgesia (Table 1). These differences provide the basis for comparing intervention direction and mechanistic level across the disease-specific sections.

**Table 1:** Representative preclinical studies of TCM-related interventions and cGAS-STING modulation across central nervous system disorders.

Disease context	Principal reported direction (descriptive)	Principal level of action	Representative intervention(s)
Cerebral ischemia or chronic hypoperfusion: upstream stress	Inhibition	Mitochondrial injury and mtDNA release; p53-related inflammation in the Buyang Huanwu Decoction study	Tetramethylpyrazine (19) Icariside II (TPI1) (76) quercetin–Fe^3+^ nanozyme (77) Buyang Huanwu Decoction (78)
Ischemic or hemorrhagic stroke: pathway and inflammatory outputs	Inhibition	cGAS–STING-related neuroinflammation, blood–brain barrier injury, ferroptosis, or autophagy	Puerarin (79) Geniposide (80) Astragaloside IV (83) Ginsenoside Rd (82)
Cerebral ischemia–reperfusion: electroacupuncture	Inhibition	Cortical cGAS–STING–NLRP3 and ferritinophagy-related signaling	Electroacupuncture at governor-vessel points (84, 85)
Alzheimer’s disease and amyloid-related cognitive models	Inhibition	cGAS–STING-related neuroinflammation, cell death, and cognitive dysfunction	Icariin (98) Tetrahydroxystilbene glucoside (100) Bisdemethoxycurcumin (101) Quercetin (102)
Age-, inflammation-, or cognitive impairment-related models	Inhibition	Study-specific mitochondrial, STING-trafficking, or NLRP3-related readouts; Huanglian Jiedu Decoction: multiomics and pharmacological STING testing	Resveratrol (107) Silibinin (106) Senegenin (104) Huanglian Jiedu Decoction (105)
Acute sleep deprivation	Restoration	Hippocampal STING signaling; H-151 attenuates protection	Resveratrol (67)
Parkinsonian models: herbal compounds or formula	Inhibition	α-Synuclein, damaged-DNA clearance, and cGAS–STING–IFN-I signaling	Astragaloside IV (116) Dingzhen Pills (117) Protocatechuic acid (118)
Parkinsonian model with cognitive impairment: acupuncture	Inhibition	Pathway involvement tested by cGAS overexpression	Acupuncture (119)
Depression and chronic stress: herbal compound or formula	Inhibition	mtDNA–microglial cGAS–STING–NLRP3 signaling	Monotropein (125) Jiao-tai-wan (jatrorrhizine) (126) Zhi-Zi-Hou-Po Decoction (127)
Depression and chronic stress: electroacupuncture	Inhibition	Pathway involvement supported by cGAS-shRNA	Electroacupuncture (120)
HSV encephalitis or HSV susceptibility: restoration of antiviral responses	Activation/restoration	cGAS–STING–IFN-I antiviral signaling	Luteolin (131) Rosmarinic acid (132)
HSV encephalitis or sepsis-associated encephalopathy: inflammatory injury	Inhibition	STING-related inflammatory output, degradation, or microglial pyroptosis	Resveratrol (134) Gelsevirine (133)
Spinal cord injury: pharmacological or delivery-system intervention	Suppression of injury-related output	Microglial inflammation, efferocytosis, autophagy, or pyroptosis	Celastrol-loaded layered double-hydroxide nanoparticle (136) Dihydromyricetin (137)
Acute postoperative pain: electroacupuncture	Activation	STING–IFN-I activation associated with analgesia and reduced neuroinflammation	Electroacupuncture at ST36/SP6 (139)
Cancer-induced bone pain: electroacupuncture	Activation	Spinal STING–IFN-I markers; Iba1/NeuN colocalization; pharmacological testing with C-176 and cGAMP	Electroacupuncture at L3–L5 Jiaji points (140)
Postoperative cognitive dysfunction	Inhibition	Celastrol: hippocampal cGAS–STING-related inflammation and pyroptotic readouts; electroacupuncture: hippocampal signaling with predominantly neuronal cGAS localization	Celastrol (141) Electroacupuncture (142)
Tumor-DNA-related neuroinflammation	Inhibition	Microglial STING signaling triggered by tumor-derived DNA	α-Cyperone (143)
Retinal neuroinflammation models	Inhibition	Alu RNA- or mtDNA-related cGAS–STING activation	EGCG or resveratrol (144) Cryptotanshinone (145)

Directions summarize the reported findings; probabilities of efficacy require evaluation in dedicated studies.

### 4.1 Ischemic stroke and cerebral ischemia–reperfusion injury

Cerebral ischemia and reperfusion consistently pair tissue protection with mitochondrial stabilization and lower mtDNA release. Ischemic oxidative stress drives mtDNA into the cytosol and activates microglial cGAS-STING (68–71). NF-κB activity and type I interferon signaling rise in parallel. The resulting response is associated with neuronal loss and blood–brain barrier injury; infarct expansion follows in severe models (72–74). HDAC3 regulation of a p65-cGAS-STING node places transcriptional control upstream of this response (75).

Mechanistic studies in cerebral ischemia place mitochondrial injury upstream of STING. Tetramethylpyrazine preserves mitochondrial dynamics and integrity in permanent cerebral ischemia and oxygen/glucose deprivation-reoxygenation models. These improvements accompany changes in mitochondrial DNA content and reduced STING activation and TBK1 phosphorylation (19). Icariside II binds triose phosphate isomerase 1 and restores mitochondrial cristae after middle cerebral artery occlusion. This change improves oxidative phosphorylation and suppresses microglial mtDNA-STING signaling (76). A mitochondria-targeted quercetin-Fe^3+^ nanozyme reaches the same upstream node by scavenging reactive oxygen species (77). Buyang Huanwu Decoction is associated with lower p53/cGAS/STING protein levels and improved cognition in chronic hypoperfusion. In oxygen–glucose-deprived BV2 cells, the p53 activator Nutlin-3 counteracts its protective and anti-inflammatory effects, supporting an upstream p53-related mechanism (78).

Other interventions act at the sensor, adaptor, or downstream outputs. Molecular docking predicts an interaction between puerarin and cGAS (79). Geniposide is associated with lower cGAS-STING and inflammatory readouts after stroke (80). In chronic cerebral hypoperfusion, resveratrol reduces STING-pathway activation and IRF3 nuclear translocation, accompanied by lower inflammatory responses (81). Studies of subarachnoid hemorrhage provide more direct pathway-perturbation results. A STING agonist reverses the antiferroptotic effect of ginsenoside Rd (82). In the astragaloside IV study, STING knockout reduces early brain injury after subarachnoid hemorrhage. Astragaloside IV treatment also lowers pathway-related inflammation and improves neurological outcomes (83).

Electroacupuncture studies concentrate on cerebral ischemia. Stimulation at governor-vessel points reduces cortical cGAS/STING signaling and NLRP3 activation after ischemia–reperfusion (84). Other models connect cognitive improvement with ferritinophagy regulated through cGAS-STING or the FTO-YTHDF1 axis (85, 86). The current experiments establish pathway involvement; neural afferent routes and responding cell populations define the next mechanistic level.

### 4.2 Alzheimer’s disease, cognitive impairment, and brain aging

In Alzheimer’s disease, amyloid-β and hyperphosphorylated tau compromise mitochondrial integrity and release mtDNA for microglial cGAS sensing (87, 88). PQBP1 recognizes extracellular tau in microglia and recruits cGAS, activating STING-related inflammation (89). In ALS models, TDP-43 induces mtDNA release through the mitochondrial permeability transition pore and activates cGAS-STING-dependent inflammatory signaling, including in patient-derived motor neurons (11).

Recent reviews link cGAS-STING/IFN-I activity to microglial activation and inflammatory cytokine expression in Alzheimer’s disease and dementia (90, 91). In 5 × FAD mice, cGAS deficiency reduces glial inflammation and amyloid-β burden. The same study links microglial cGAS to neurotoxic A1 astrocyte responses (92). In a separate Alzheimer’s disease model, STING inhibition reduces microglial synapse engulfment and associated pathology (93). Reviews of CNS senescence describe an additional connection between intracellular stress and senescence-associated secretory inflammation, with cGAS-STING among the implicated pathways (94). APOE and TREM2 shape disease-associated microglial states (95, 96); their intersection with cGAS-STING is a defined target for cell-specific experiments.

Human studies currently provide mainly associative findings. TET2 loss-of-function variants are associated with faster cognitive decline in amyloid-positive individuals, whereas Tet2-deficient mice show increased cGAS-STING activity and pathology that is partly reversed by antioxidant treatment (97). These results support pathway involvement in brain aging-related inflammation; dedicated human studies are needed to define its independent contribution to cognitive decline.

TCM-related studies in Alzheimer’s disease models focus on downstream inflammatory signaling. Icariin reduces microglial cGAS-STING responses in an Aβ1-42 model; the study also uses the cGAS inhibitor RU.521 to examine pathway involvement (98). The flavonoid fraction of Epimedium brevicornu has also been associated with reduced microglial cGAS-STING responses (99). Tetrahydroxystilbene glucoside lowers cGAS-STING activity and NLRP3 signaling, with inhibitor experiments included in the study (100). Bisdemethoxycurcumin reduces RIPK/MLKL-dependent necroptosis (101). Quercetin has been linked to the pathway through estrogen-receptor signaling (102, 103). Senegenin was examined through network pharmacology and cellular validation (104). The Huanglian Jiedu Decoction study combined machine-learning-guided multiomics with pharmacological STING testing using H-151 and DMXAA (105). These designs assign different levels of mechanistic confidence.

A separate group of studies targets mitochondrial quality control or STING trafficking. Silibinin restores mitochondrial homeostasis in LPS-stimulated microglia and lowers STING signaling through a TREM2-related mechanism (106). Resveratrol blocks STING movement from the ER to the Golgi, reducing subsequent TBK1 activation and microglial senescence (107). In acute sleep deprivation, resveratrol instead partly restores reduced hippocampal STING signaling while suppressing neuronal ferroptosis and improving cognition. H-151 partly weakens its neuroprotective effects (67). These experiments were performed in cells or rodents. Reviews of immune imbalance in Alzheimer’s disease discuss broader inflammatory mechanisms and candidate compounds such as salidroside and magnolol (108). Curcumin and EGCG are covered in a review of phytochemical interventions in brain aging and neurodegeneration that integrates findings from experimental studies (109). These secondary sources provide a broader phytochemical context; cGAS-STING involvement should be assessed from the experiments reported for each compound.

Preclinical models of Alzheimer’s disease and brain aging consistently pair neuroprotection with lower cGAS-STING inflammatory output. Measurements of immune surveillance and abnormal-protein clearance will determine how far pathway suppression can be taken without disrupting homeostatic functions.

### 4.3 Parkinson’s disease and *α*-synucleinopathies

A proposed framework for Parkinsonian injury places mitochondrial dysfunction and α-synuclein-associated stress upstream of cGAS-STING-related inflammation (110–112). Reviews of the pathway connect these processes with glial inflammation and dopaminergic injury across experimental settings (113, 114). Constitutively active STING is sufficient to initiate α-synuclein pathology in mice, with substantia nigra inflammation preceding neuronal loss (9). DJ-1-Nrf2 suppresses STING and connects mitochondrial homeostasis to control of this inflammatory loop (115).

Parkinson’s disease studies use two preclinical strategies: suppression of STING-related inflammation and clearance of damaged DNA. Astragaloside IV reduces neuronal loss and motor deficits in rotenone and mutant α-synuclein models. Agonist and inhibitor experiments place α-synuclein aggregation upstream of a γH2AX-STING-IFN-I sequence (116). Dingzhen Pills reduce cGAS-STING readouts in MPTP-treated mice and are accompanied by lower neuronal ferroptosis and neuroinflammation; the design used pathway readouts without a dependence experiment (117).

Protocatechuic acid acts mainly on upstream DNA clearance. It enhances autophagy in microglia, promotes clearance of damaged DNA, and reduces STING activation. Microglia-specific Atg5 loss abolishes the benefit, which is also accompanied by reduced α-synuclein aggregation in A30P mice (118). Acupuncture likewise reduces cGAS-STING signaling and improves cognition in 6-OHDA-treated rats. cGAS overexpression reverses this improvement, supporting pathway involvement; an independent binding assay would be required to define any direct acupuncture-cGAS interaction (119).

In Parkinsonian models, benefit follows lower cGAS-STING activity or improved damaged-DNA clearance. Pharmacological perturbation and cGAS overexpression establish pathway involvement for selected interventions. Cell-specific Atg5 loss identifies autophagy as an upstream requirement. This ordering separates mitochondrial quality control from direct action on cGAS or STING.

### 4.4 Depression and stress-related neuroinflammation

Stress models differ in their initiating insult but converge on mitochondrial injury and mtDNA release. Microglial cGAS-STING activation then accompanies neuroinflammation and depressive-like behavior (54, 120–122). Ferroptotic responses add a cell-death component to this sequence (123). In chronic restraint stress, inhibition of P2X7R or STING reduces neuroinflammation and depressive-like behavior. The study also examines LPS-stimulated BV2 microglia, in which P2X7R knockdown or inhibition attenuates mitochondrial injury and mtDNA release. STING/IRF3 phosphorylation also declines in these cells (124). These results support the proposed upstream ordering in the tested restraint-stress and LPS-BV2 settings.

Available TCM-related studies consistently associate benefit with reduced STING signaling, but the implicated levels range from mitochondrial homeostasis and mtDNA release to STING activity and downstream inflammatory output. Monotropein, derived from Morinda officinalis, lowers cGAS and phosphorylated STING levels and reduces microglial activation in mice with LPS-induced depressive-like behavior. Co-administration of the STING inhibitor C-176 was reported to attenuate its anti-inflammatory effect (125). Jiao-tai-wan likewise attenuates STING-related signaling in a chronic restraint-stress model. Component analysis identified jatrorrhizine as one candidate active constituent, while microglial STING knockdown supports pathway involvement in the formula’s anti-inflammatory and behavioral effects (126).

Zhi-Zi-Hou-Po Decoction acts mainly at the upstream level of mitochondrial quality control. It restores neuronal TFAM-dependent mitophagy and promotes clearance of damaged mitochondria and cytosolic mtDNA, thereby reducing subsequent cGAS-STING activation; TFAM knockdown abolishes this effect (127). Conditioned medium from treated neurons also shifts microglia toward an anti-inflammatory phenotype, linking neuronal mitochondrial homeostasis to altered neuron–microglia communication.

Electroacupuncture studies have focused mainly on downstream cGAS-STING inflammatory output. In a chronic unpredictable mild-stress model, electroacupuncture suppresses cGAS-STING-NLRP3 signaling and restores hippocampal monoamine levels (120). Hippocampal cGAS-shRNA produces a similar antidepressant effect, identifying cGAS as a relevant regulatory node in this model. Direct interaction testing between electroacupuncture and cGAS perturbation would further define the pathway’s contribution to the overall effect.

### 4.5 Viral encephalitis and sepsis-associated encephalopathy

Infection creates two therapeutic targets. Early HSV-1 infection requires restoration of cGAS-cGAMP-STING-IFN-I defense. Later virus-associated injury and sepsis favor restriction of sustained inflammatory output. Canonical signaling establishes an antiviral state in neural cells, so indiscriminate blockade can weaken host defense (128, 129). Non-cGAMP cGAS functions and persistent inflammation contribute separately to encephalitic injury (14–16, 130).

Luteolin and rosmarinic acid restore the early antiviral response at different pathway levels. Luteolin binds the substrate pocket of cGAS and promotes oligomerization. Type I interferon production rises, and HSV-1 replication falls in cellular and murine encephalitis models (131). Rosmarinic acid acts on STING trafficking. Stress-induced ALOX15 activity generates 4-HNE, which modifies STING and blocks its movement from the ER to the Golgi. Rosmarinic acid inhibits this sequence and restores antiviral signaling (132). Both interventions correct a suppressed response within the early infection window.

When inflammatory injury dominates infection, STING inhibition is protective. In sepsis-associated encephalopathy, microglial STING activation drives pyroptosis. Gelsevirine lowers STING phosphorylation and improves survival and cognition. Its suppression of microglial pyroptosis is weakened after STING knockout, and its protective effects are lost, supporting a contribution of STING to the treatment response (133). Resveratrol targets host inflammation in HSV-1 encephalitis. It disrupts the HSP90β-STING interaction and promotes STING degradation, reducing proinflammatory microglial responses (134). Viral clearance was not the measured mechanism in that study.

Treatment direction changes as infection progresses. Luteolin and rosmarinic acid restore early antiviral signaling. Gelsevirine and resveratrol restrict inflammatory tissue injury after sustained pathway activation. Designs that measure viral load alongside interferon activity can define the transition between these targets; neural injury provides the corresponding safety endpoint.

### 4.6 Pain and spinal cord injury

Pain models show context-dependent neuronal and glial cGAS-STING responses. Transient STING-IFN-I signaling in peripheral sensory neurons suppresses ion-channel activity and reduces excitability. Pathological activation in the spinal cord instead drives microglial cytokine release and central sensitization (129). Peripheral nerve injury also releases spinal mtDNA and sustains an IRF3-CMPK2 feedback loop in microglia; CMPK2 silencing reduces pain (135). These findings implicate both neuronal excitability and spinal immune responses in pathway-related pain modulation.

Traumatic spinal cord injury combines mitochondrial oxidative stress with defective clearance of apoptotic cells (136). Celastrol-loaded layered double hydroxide nanoparticles scavenge reactive oxygen species and preserve mitochondrial homeostasis. They also improve microglial clearance of apoptotic debris, reducing cGAS-STING signaling during functional recovery (136). Dihydromyricetin inhibits microglial STING and restores autophagy. STING overexpression reverses its effect on NLRP3-related pyroptosis (137). Mitochondrial protection and phagocytic clearance act upstream by reducing the DNA burden that sustains STING inflammation.

Electroacupuncture research includes both spinal cord injury and postoperative pain. Electroacupuncture at Jiaji points (EX-B2) reduces neutrophil extracellular trap formation after spinal cord injury. The study reports lower spinal cGAS and STING protein expression, accompanied by reduced neuroinflammation and glial scarring. DNase I treatment supports a contribution of extracellular DNA to these changes; cGAS and STING were measured without node-specific perturbation (138). In an acute postoperative pain model, electroacupuncture at ST36 and SP6 increases the pain threshold and reduces neuroinflammation while restoring STING-IFN-I signaling. Intrathecal C-176 weakens its analgesic and anti-inflammatory effects (139).

Electroacupuncture at L3-L5 Jiaji points also increases spinal STING and IRF3 expression in cancer-induced bone pain. IFNAR and IFN-α/β measures rise, while TNF-α and IL-1β fall during analgesia. STING immunoreactivity is predominantly colocalized with Iba1-positive microglia and, to a lesser extent, NeuN-positive neurons. C-176 partly attenuates electroacupuncture-induced analgesia (140). The same study reports analgesia with cGAMP and with C-176 alone. cGAMP increases proinflammatory cytokines that decline after electroacupuncture, indicating that analgesia and anti-inflammatory effects should be interpreted separately (140).

Most acupuncture and electroacupuncture models with explicit cGAS-STING readouts show lower inflammatory output. The acute postoperative pain and cancer-induced bone pain studies show that this improvement can accompany increased STING-IFN-I signaling (Table 2). Changes in inflammatory markers should therefore be distinguished from the direction of pathway modulation. Figure 3 summarizes the acupuncture findings and the proposed connection between somatosensory input and neural-cell responses.

**Table 2:** Representative preclinical studies of acupuncture and electroacupuncture involving cGAS-STING signaling or upstream DNA-related injury.

Intervention and acupoints, when reported	Disease model	Reported direction	Mechanistic basis linking the intervention to cGAS-STING	Reference(s)
Electroacupuncture (governor-vessel points)	Cerebral ischemia–reperfusion/MCAO	Inhibition	Reduced cortical cGAS/STING/NLRP3; modulation of cGAS–STING-related ferritinophagy	(84, 85)
Acupuncture	6-OHDA-induced Parkinsonian model with cognitive impairment	Inhibition	Reversal by cGAS overexpression supports pathway involvement	(119)
Electroacupuncture	Chronic unpredictable mild stress (depression)	Inhibition	Genetic perturbation: cGAS-shRNA reproduces part of the benefit and supports pathway involvement; it does not show that cGAS mediates the entire electroacupuncture effect	(120)
Electroacupuncture (ST36 and SP6)	Acute postoperative pain	Activation	Restored STING–IFN-I signaling; C-176 weakened electroacupuncture-induced analgesia and anti-inflammatory effects. This supports pathway involvement, without demonstrating direct action on STING.	(139)
Electroacupuncture (L3–L5 Jiaji)	Cancer-induced bone pain	Activation	Increased STING–IFN-I-related markers; Iba1/NeuN colocalization; C-176 partly attenuated electroacupuncture analgesia; cGAMP also produced analgesia	(140)
Electroacupuncture	Postoperative cognitive dysfunction	Inhibition	Reduced hippocampal cGAS–STING-related inflammation; predominantly neuronal cGAS localization; systemic RU.521 add-on testing	(142)
Electroacupuncture (Jiaji points, EX-B2)	Spinal cord injury	Lower expression	cGAS/STING western blot and NET-related measurements; DNase I intervention; no cGAS- or STING-specific perturbation	(138)

**Figure 3:**
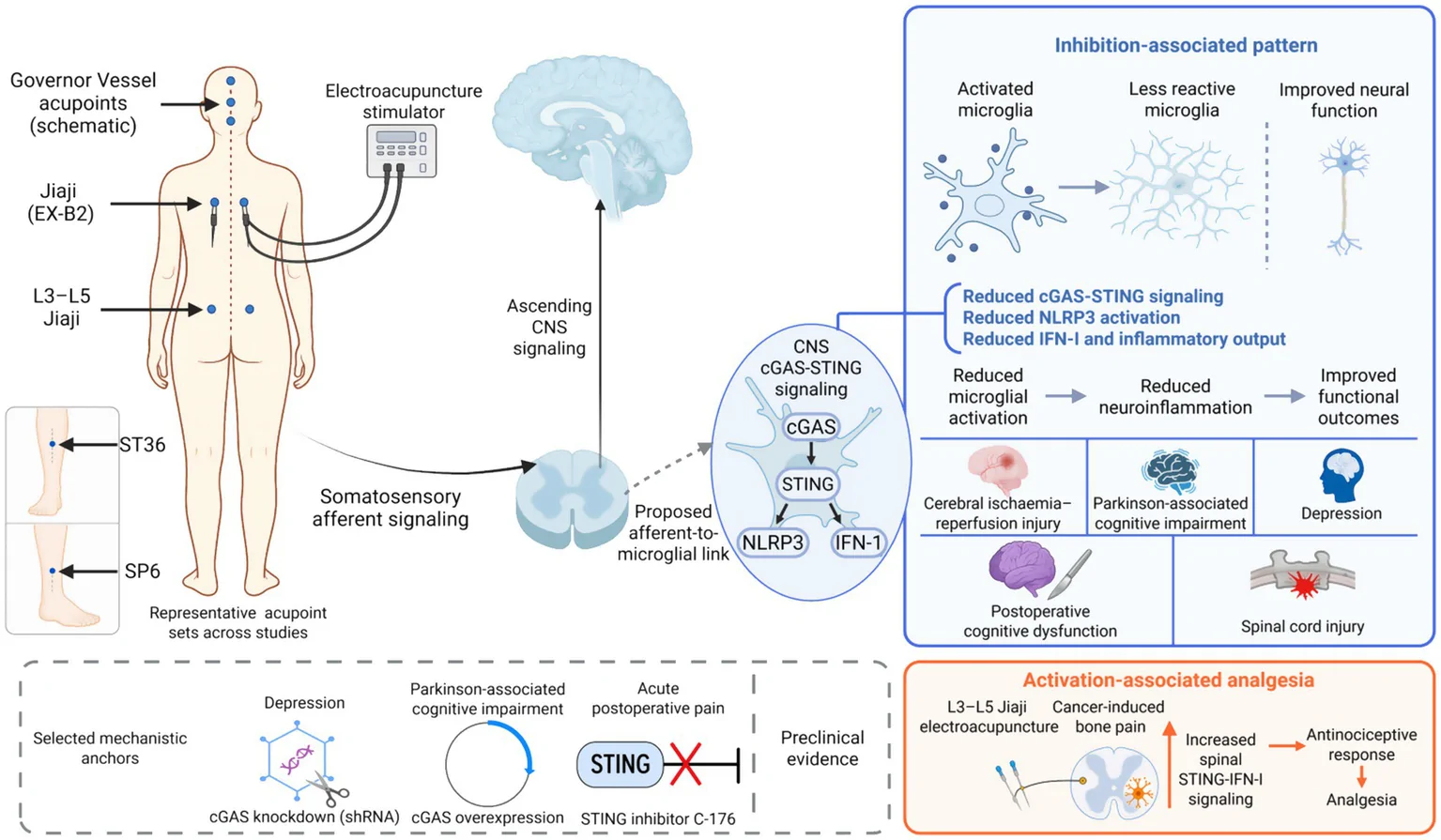
Preclinical associations of acupuncture and electroacupuncture with cGAS-STING signaling in the central nervous system. Representative governor-vessel points, L3-L5 Jiaji points, ST36, and SP6 generate somatosensory input. The studies summarized include explicit cGAS-STING measurements and genetic or pharmacological interventions in selected models. The EX-B2 spinal cord injury study measures cGAS/STING protein expression and uses DNase I to examine upstream DNA- and NET-related injury. The link from afferent input to defined neural-cell responses remains to be tested. Acute postoperative pain is shown as a mechanistic anchor involving pharmacological STING inhibition, whereas the cited cancer-induced bone pain model associates electroacupuncture-induced analgesia with increased spinal STING-IFN-I signaling. Blue and orange denote inhibition-associated and activation-associated patterns, respectively.

### 4.7 Other sterile neuroinflammatory conditions

Postoperative cognitive dysfunction and retinal neuroinflammation involve different responding cell populations. Celastrol reduces hippocampal cGAS-STING-related inflammation and pyroptotic readouts in postoperative models. The STING agonist DMXAA counteracts its protective effects (141). In aged rats undergoing splenectomy, electroacupuncture reduces hippocampal cGAS-STING signaling, with cGAS predominantly localized to neurons (142). In a separate model using Lewis tumor-cell DNA to stimulate microglia, α-cyperone suppresses STING-related inflammatory responses. STING siRNA reduces inflammatory signaling in that model, while DMXAA counteracts the siRNA effect (143). In retinal pigment epithelial cells, EGCG and resveratrol each reduce Alu RNA-induced interferon responses and cell death (144). Cryptotanshinone reduces mtDNA-related signaling and binds STING directly (145). These findings need to be interpreted within their respective cellular and injury settings.

The disease sections identify model-specific patterns of modulation. Sterile injury models include protection associated with either reduced or restored signaling. Early infection requires restoration of suppressed defense. Pain studies require interpretation of cellular responses together with the functional effects of pathway perturbation. The next section compares where each intervention enters the pathway.

## 5 Mechanisms of cGAS-STING modulation by TCM-related interventions

TCM-related interventions enter the pathway at four levels (Table 3). Some reduce the cytosolic DNA burden. Others act on cGAS or STING, alter STING trafficking, or restrict downstream inflammatory and cell-death output. This ordering separates signal input from pathway involvement and downstream response.

**Table 3:** Reported levels of action, study types, and mechanistic basis of TCM-related interventions on the cGAS-STING axis.

Reported or proposed level of action	Proposed or observed change	Representative intervention(s)	Mechanistic basis for pathway placement	Key reference(s)
① Upstream mitochondrial protection	Preserve mitochondrial integrity or cristae and reduce associated STING signaling	Tetramethylpyrazine; Icariside II (TPI1)	Tetramethylpyrazine: mitochondrial and STING/TBK1 readouts; Icariside II: TPI1-related cristae remodeling and mtDNA–STING responses	(19, 76)
① Upstream mitochondrial protection	Reduce oxidative stress and mtDNA release	Quercetin–Fe^3+^ nanozyme	Oxidative-stress and mtDNA-release measurements	(77)
① Upstream clearance or stress control	Promote mitochondrial quality control or attenuate upstream stress signaling	Buyang Huanwu Decoction; Zhi-Zi-Hou-Po Decoction	Buyang Huanwu: Nutlin-3/p53 activation counteracts protection in OGD-BV2 cells; Zhi-Zi-Hou-Po: TFAM knockdown tests upstream mitochondrial quality control, not direct STING perturbation	(78, 127)
② Reported cGAS interaction	Potential sensor interaction	Puerarin; Luteolin	Molecular docking for puerarin and biochemical binding for luteolin; these findings do not establish target engagement or pathway necessity	(79, 131)
② Reported STING interaction	Alter adaptor activity	Cryptotanshinone	Direct biochemical binding SPR and mutational analysis; direct binding is not equivalent to necessity for tissue protection	(145)
③ STING trafficking or degradation	Reduced signaling associated with HSP90β-dependent degradation or ER–Golgi blockade	Resveratrol	Protein-interaction and trafficking analyses	(107, 134)
③ STING degradation	Promote lysosomal clearance	Artesunate	Lysosomal-function analysis	(146)
③ Restoration of impaired STING trafficking	Relieve lipid-peroxidation-related suppression of antiviral signaling	Rosmarinic acid	4-HNE adduct and STING-trafficking analyses	(132)
④ Downstream output: cognitive models	Reduce cGAS–STING-related inflammatory output	Icariin; Tetrahydroxystilbene glucoside	Icariin: cGAS inhibitor RU.521; tetrahydroxystilbene glucoside: cGAS–STING inhibitor experiments reported	(98, 100)
④ Downstream output: stroke	Reduce inflammatory, autophagy-related, or cell-death output	Geniposide; Astragaloside IV	Geniposide: pathway readouts; astragaloside IV study: STING knockout tests SAH injury, alongside drug-associated pathway and inflammatory readouts	(80, 83)
④ Downstream output: depression or sepsis	Reduce neuroinflammation or pyroptotic output	Monotropein; Gelsevirine	Monotropein: C-176 co-administration reportedly attenuates anti-inflammatory effects; gelsevirine: protective effects are lost after STING knockout	(125, 133)
④ Downstream output: spinal cord injury	Inhibit STING, restore autophagy	Dihydromyricetin	STING overexpression and autophagy-related readouts	(137)
④ Downstream output: postoperative or tumor-DNA-related injury	Reduce STING-related inflammatory output	Celastrol; α-Cyperone	Celastrol: DMXAA counteracts protection; α-cyperone study: STING siRNA lowers inflammatory signaling and DMXAA counteracts the siRNA effect	(141, 143)

### 5.1 Mitochondrial protection and reduction of cytosolic DNA burden

Damaged mitochondria are a recurrent source of activating DNA in sterile central nervous system models. Mitochondria-ER contact sites modify the transfer of this stress signal to STING (42, 43). Tetramethylpyrazine and icariside II improve mitochondrial readouts, while mitochondria-targeted quercetin nanozymes reduce oxidative stress and mtDNA release (19, 76, 77). Zhi-Zi-Hou-Po Decoction restores mitochondrial quality control and mtDNA clearance (127). Buyang Huanwu Decoction is linked to lower p53/cGAS/STING signaling (78). Upstream control reduces the stimulus before direct sensor inhibition becomes necessary, offering a mechanistically distinct route for sterile injury.

### 5.2 Direct modulation of cGAS and STING

Direct-action claims differ sharply by study design. Molecular docking places puerarin near cGAS and defines a binding hypothesis (79). Biochemical assays show that luteolin binds cGAS and promotes antiviral signaling in an HSV model (131). Surface plasmon resonance and mutational analysis establish cryptotanshinone binding to STING (145). In retinal pigment epithelial cells, pull-down assays detect binding of EGCG and resveratrol to G3BP1, a regulator of cGAS activation. These assays concern G3BP1 binding rather than direct binding to cGAS (144).

Pathway perturbation provides a second level of mechanistic attribution. Knockdown or knockout can test pathway contributions to injury; comparing treatment responses under the same perturbation addresses involvement in drug action. Overexpression and agonist experiments assess whether increasing signaling counteracts protection. These designs have been used in stroke and Parkinsonian models (83, 119). Depression and sepsis studies also include targeted perturbation (120, 133). Spinal cord injury and acupuncture studies provide additional examples (137, 139). Interpretation depends on whether the experiment tests the injury mechanism, the intervention response, or both.

### 5.3 Regulation of STING trafficking and degradation

STING initiates signaling after moving from the ER to the Golgi and is later cleared through the endolysosomal system. Trafficking determines signal onset; degradation controls its duration (32). This level can restrict persistent inflammation or restore antiviral signaling when the pathway is suppressed.

Resveratrol blocks ER-Golgi trafficking and can disrupt the HSP90β-STING interaction, promoting STING degradation (107, 134). Artesunate restores lysosomal clearance (146). Rosmarinic acid acts in the opposite infection context: it reduces 4-HNE modification and restores STING trafficking (132). Trafficking control can suppress persistent inflammation or recover an antiviral response, depending on the initial pathway state.

### 5.4 Downstream inflammatory and cell-death pathways

TBK1-IRF3 and NF-κB are the most common signaling outputs in the reviewed studies. NLRP3 activation is often measured alongside them (62, 65). Cell-death readouts most often concern ferroptosis and pyroptosis. Necroptosis is measured in a smaller group of models. These markers describe the inflammatory state across disease models, but they do not by themselves establish that an intervention acts through cGAS or STING.

Downstream marker reduction is compatible with several mechanisms. Antioxidant activity can produce it, as can broad anti-inflammatory action or altered autophagy. Changes in treatment response after STING knockout or cGAS knockdown provide functional support for pathway involvement. Overexpression and pharmacological reversal experiments further assess the response to altered signaling. Direct binding assays test molecular interaction. Cell-specific localization identifies where the measured signal occurs. Protein expression or phosphorylation alone establishes pathway association, not direct action on cGAS or STING.

Mechanistic studies fall into three levels. Associated readouts show that pathway activity changes with treatment. Genetic or pharmacological perturbation establishes pathway involvement. Independent biochemical assays define direct action. Upstream DNA control explains signal input, and STING trafficking explains signal duration. Clinical value requires separate efficacy, exposure, and safety assessment.

## 6 Discussion and future directions

The combined literature supports a context-sensitive framework for comparing cGAS-STING modulation across pathological process, responding compartment, and site of intervention. Reduced signaling accompanies benefit in several sterile injury models, whereas the sleep-deprivation study reports restoration of reduced STING signaling (67). Early HSV infection is associated with restoration of suppressed defense signaling. Pain studies report both antinociceptive and inflammatory responses to STING signaling. These findings organize herbal and acupuncture studies by disease stage and cellular source; they do not establish a universal direction of modulation.

Figure 2 is a disease-context model, not a measured therapeutic threshold. Directional differences occur both across and within disease categories. Treatment timing and signal duration are candidate explanations for this variation. Cell identity is less often resolved because many studies analyze whole brain regions or spinal cord tissue. Cell-specific perturbation will determine which neuronal, glial, or peripheral immune populations control each response.

Study design determines the strength of mechanistic attribution. Parallel marker changes establish pathway association. Knockout or knockdown tests necessity, and overexpression or pharmacological rescue tests functional involvement. Direct physical action requires an independent binding assay. Puerarin is currently supported by molecular docking; luteolin and cryptotanshinone have biochemical binding results (79, 131, 145). Pathway change and direct target engagement are separate conclusions.

Clinical translation depends on effective exposure. Polyphenol absorption and metabolism reduce systemic availability, while blood–brain barrier passage determines the concentration that reaches neural tissue (147). Experimental inhibitors require the same accounting for brain penetration and off-target activity (148). Delivery systems can improve distribution, but the carrier and its surface properties also change cellular uptake. Protein-corona formation adds an immune variable. Free-compound, empty-carrier, and loaded-system controls can separate these effects, with tissue exposure measured alongside pathway readouts (77, 136, 149).

Formula studies need chemical composition and *in vivo* exposure data before individual constituents can be assigned a mechanistic role. Network pharmacology and multiomics can nominate candidates; constituent identification and pharmacokinetics establish which candidates reach the relevant tissue. Perturbation experiments test functional involvement at the node actually manipulated (78, 127). In the Huanglian Jiedu Decoction study, multiomics prioritized the pathway, and separate pharmacological experiments tested STING-related responses (105). Acupuncture reports should specify acupoint location and stimulation frequency. Current intensity, session duration, and treatment course require separate reporting. Randomization and blinding should be reported alongside sample-size justification and appropriate controls (150). Cell localization is also needed to distinguish neural-tissue responses from peripheral immune-cell responses. Within neural tissue, neuronal and glial responses require separate localization.

Human studies currently document disease-associated pathway changes, while intervention studies are predominantly preclinical (97, 151). Translation requires trials that define the responding brain cell population and treatment window before testing efficacy. Publication is also weighted toward positive findings. Prospective protocols, complete outcome reporting, and independent replication can reduce selective reporting. Standardized effect sizes will make results comparable across models and interventions.

A decisive experiment must connect intervention, pathway perturbation, and functional outcome in the same model. Cell-specific manipulation can identify the responding compartment, and repeated time points can show when the direction changes. Exposure is the relevant endpoint for natural compounds; viral load is essential in infection; tissue injury anchors sterile models. Formula and acupuncture studies also need minimum reporting standards that permit replication. This validation chain will define the benefit–risk balance for each indication.

## 7 Conclusion

cGAS–STING offers a useful framework for comparing TCM-related interventions across neuroinflammatory disorders. Within sterile injury, protection has been reported with suppression of excessive signaling or restoration of reduced signaling. Early viral infection is associated with restoration of defense signaling, whereas pain models show both antinociceptive and inflammatory effects of STING signaling. Current studies address cytosolic DNA control, sensor modulation, STING trafficking, and downstream inflammatory output. Their experimental support ranges from pathway association to perturbation and direct-binding results, which answer different mechanistic questions. Most findings remain preclinical and model-dependent. Defined cellular targets, treatment windows, brain exposure, and safety will determine which interventions merit clinical testing.
